# Uncertainty about lithium and suicide: A response to Nassir Ghaemi

**DOI:** 10.1177/02698811251346706

**Published:** 2025-06-17

**Authors:** Joanna Moncrieff, Jacki Stansfeld, Lisa Wood, Martin Plöderl

**Affiliations:** 1 University College London, London, UK; 2 North East London NHS Foundation Trust, Rainham, UK; 3 Paracelsus Medical University, Salzburg, Austria

We are grateful for the opportunity to respond to Nassir Ghaemi’s article (Ghaemi, 2024) about our review of randomised trials of lithium’s effects on suicide (Nabi et al., 2022).

We had already addressed Ghaemi’s main points in a reply to another comment about our review (Bschor et al., 2022). However, since Ghaemi does not cite this, we summarise our response to these points here.

There are two main reasons why we chose to conduct a review of lithium and suicidality and to update the findings of the influential review by Cipriani et al. (2013).

First, conventions about how to manage trials in which there are zero events (suicides in this case) have changed. It has been increasingly recognised that excluding them, as was done by Cipriani et al. (2013), is problematic for various reasons, including those outlined by Diamond et al. (2007) and cited by Ghaemi. This is because excluding such studies can produce a misleading event rate and gives small studies with events undue weight in relation to all existing data. The fact that a small number of suicides have occurred in some lithium trials does not invalidate these concerns, as Ghaemi claims it does.

Second, a large, high-quality trial, specifically designed to test the anti-suicidal properties of lithium, was published in 2021 (Katz et al., 2022). This did not show evidence that lithium had preventive effects on suicide-related events (including suicide), which could influence the results of meta-analysis.

We agree with Ghaemi that suicide is a difficult outcome to study because it is rare, and therefore, demonstrating the effect of any intervention is challenging. Moreover, it may not be reliably reported. Suicide attempts happen about 20 times more frequently than suicides, and thus, the trial data have greater power to detect a preventive effect for lithium. Although we partly agree with Ghaemi on differences between fatal and nonfatal suicidal behaviour, and evidence suggests the aetiologies of these events are ‘incompletely overlapping’ (Edwards et al., 2021), a suicide attempt is the single most important predictor of suicides. Thus, it is a common surrogate in suicide research and an important target in suicide prevention (World Health Organization, 2014). If lithium does prevent suicide, we would likely expect to see a preventive effect on suicide attempts, yet the evidence does not support a preventive effect of lithium on suicide attempts in any recent meta-analyses, including that of Cipriani et al. (2013).

We also agree that meta-analysis is an imperfect method, whose results are susceptible to decisions about which studies to include and exclude. The main measure that can be taken to guard against the intrusion of conscious or unconscious biases into the study selection process is the prepublication of a protocol with clear eligibility criteria. We did this (PROSPERO CRD42021265809). Although Ghaemi accuses us of ‘self-deception’ and ‘obviously biased pseudoscience’, Ghaemi is known for his belief that lithium is unique among psychiatric drugs for being effective and biologically specific, and part of his case for this point of view is that lithium prevents suicide (Ghaemi, 2024). Perhaps, this contributed to the tone and content of his paper and the selective, post hoc method of summarising the evidence.

Putting aside the ad hominem accusations and a language that is ‘outside the usual academic norms’ (Reilly, 2024), Ghaemi has two substantive criticisms of our review, which concern our eligibility criteria: the inclusion of trials with zero events and the exclusion of trials performed before the year 2000.

In the past, trials with zero events were commonly excluded from meta-analyses because of the difficulty of including them using conventional methods of meta-analysis. It is now known that this may lead to biased results, and since new methods have been developed to enable them to be included, it is widely recommended that trials with zero events should be included. In 2015, for example, Kuss recommended that ‘Methods that ignore information from double-zero studies or use continuity corrections should no longer be used’ (Kuss, 2015). Ren et al. (2019) suggested ‘To utilize all available information and reduce research waste and avoid overestimating the effect, meta-analysts should incorporate double zero studies, rather than simply removing them’. Recently, Xu et al. (2022) argued that ‘Including double-zero studies in meta-analysis improved performance substantively when compared to excluding them, especially when the proportion of double-zero studies was large’. The Cochrane Collaboration also now provides a tutorial on how to deal with data from trials with zero events and is critical about dismissing it (Xu, 2021). Of note, Peto’s traditional method is not considered suitable for the type of studies in our analysis (Xu et al., 2022). Nonetheless, we had also applied and reported non-optimal methods such as Peto’s method, but Ghaemi did not acknowledge this. All the methods we used produced the same conclusion – that we cannot say whether lithium affects suicide risk in one way or another due to a high level of uncertainty (wide confidence intervals; Nabi et al., 2022, Table 2).

Part of Ghaemi’s argument for excluding trials with zero suicides is that they consist of relapse prevention studies in people with bipolar disorder and that they were not set up to explore the anti-suicidal effects of lithium. As such they were likely to have actively excluded people with suicidal ideation. However, this is also true of some of the trials in which suicides occurred. The correct way to explore whether an anti-suicidal effect of lithium is specifically revealed in trials that involve people at high risk of suicide is to do a subgroup analysis. We did this (Table 7S in Nabi et al., 2022), and the results were inconclusive and the degree of uncertainty was high.

Ghaemi’s other criticism of our eligibility criteria is that we excluded trials conducted before 2000 in our main analysis. We did this because there is evidence that suicide was not reliably reported in earlier trials. For example, there is subsequent evidence that there was, in fact, a suicide in at least one trial in which none were reported (Glen et al., 1984). We suggest that an analysis that includes all the data from higher quality trials published from 2000 (after the CONSORT criteria had established uniform standards for trial reporting) is more reliable than one that includes a small number of trials of variable quality mostly published in the 1960s and 1970s. However, we conducted a sensitivity analysis including trials published prior to 2000 (Figure 3 in Nabi et al., 2022), and the results remained inconclusive with a high degree of uncertainty. However, Ghaemi does not mention that we performed this analysis, despite the fact that these results were highly visible in our paper and included in the abstract.

Ghaemi also criticises our classification of suicides in the large suicide prevention trial by Katz et al. (2022). Three deaths were reported in the placebo group in this trial, but only one was a suicide that occurred during the trial. One was an opioid overdose that was not classified as a suicide, and one was a suicide that occurred 1 month after the end of the trial (and was only detected much later). In order to be consistent with other studies (which may have found suicides that occurred after the trial and not reported them), we did not include this event, and Katz et al. (2022) did not include it in their analysis either.

Ghaemi chose to classify both events as suicide in his re-analysis. Whereas there may be a case to include the suicide that occurred after the trial, there is little justification to include the other.

Ghaemi’s main argument is that the fact that there were slightly more suicides among placebo-treated patients across trials than among people who had been allocated to lithium shows that lithium has an anti-suicidal effect, even though the numbers of suicides are small and simple power-analyses reveal that the whole database of clinical trials is clearly underpowered.^1^ He argues that the difference is meaningful even though we showed it was not statistically significant and, therefore, could have occurred by chance.

Ghaemi then conducts his own meta-analysis, without a pre-published protocol, in which he chooses to exclude trials with zero events and to include those conducted prior to 2000 that reported a suicide. This resulted in the inclusion of only six trials and produced an effect which Ghaemi highlighted as a result that can be ‘believed with over 90% confidence’. In a further analysis in which he re-classified two deaths in the Katz study as suicide, the effect was ‘statistically significant’.

Ghaemi concludes that: ‘The anti-suicide effect of lithium is supported strongly by randomised clinical trials’. This statement is clearly at odds with the randomised trial evidence on suicide when using appropriate statistical methods and taking into account the uncertainty, and with the findings for suicide attempts and the results of the large and high-quality suicide prevention trial by Katz et al. (2022). Even if we accept the validity of Ghaemi’s re-analysis, a result that depends on the subjective classification of one or two events is not a strong support for lithium’s antisuicidal properties.

This uncertainty is acknowledged by other proponents of lithium. Baldessarini and Tondo (2022), for example, recently described how: ‘recruiting participants to such trials (suicide prevention trials of lithium) may be made difficult by an evidently prevalent belief that the question of antisuicidal effects of lithium is already settled, which it certainly is not’ (Baldessarini and Tondo, 2022: 10).

We all have beliefs and make assumptions about the nature and effects of psychiatric treatments. We all need to guard against allowing these to distort our research, using methods such as a pre-specified protocol, and sensitivity analyses. Ghaemi’s post hoc selection of studies and use of an outdated analysis method suggest that his analysis is problematic.

## Notes

1. A power analysis, assuming that lithium prevents suicides by 50% (a very large effect), a rate of suicides of 0.5% in the placebo arm as seen in the trials (Figure 3 in Nabi et al., 2022), α = 0.05, power = 80% (α = 0.20) and two-sided testing, suggests that there should be 9382 patients in each arm. In all trials combined, including those before 2000, there were less than 2000 patients in each arm. R-code: power.prop.test (p2 = 0.005, p1 = 0.005/2, power = 0.80, sig.level = 0.05).
